# An Exploratory Analysis of the Role of Adipose Characteristics in Fulltime Wheelchair Users’ Pressure Injury History

**DOI:** 10.3389/fbioe.2021.753897

**Published:** 2021-11-29

**Authors:** Sharon Eve Sonenblum, Megan Measel, Stephen H. Sprigle, John Greenhalgh, John McKay Cathcart

**Affiliations:** ^1^ Rehabilitation Engineering and Applied Research Laboratory, The George W. Woodruff School of Mechanical Engineering, Georgia Institute of Technology, Atlanta, GA, United States; ^2^ Wallace H. Coulter Department of Biomedical Engineering, Georgia Institute of Technology, Atlanta, GA, United States; ^3^ College of Design, Georgia Institute of Technology, Atlanta, GA, United States; ^4^ FONAR Corporation, Melville, NY, United States; ^5^ School of Health Sciences, Ulster University, Northern Ireland, Coleraine, United Kingdom

**Keywords:** adipose, pressure injury, pressure ulcer, MRI, biomechanical risk, wheelchair, spinal cord injury

## Abstract

**Aim:** The goals of this study were 1) to identify the relationship between adipose (subcutaneous and intramuscular) characteristics and pressure injury (PrI) history in wheelchair users and 2) to identify subject characteristics, including biomechanical risk, that are related to adipose characteristics.

**Materials and Methods:** The buttocks of 43 full-time wheelchair users with and without a history of pelvic PrIs were scanned in a seated posture in a FONAR UPRIGHT® MRI. Intramuscular adipose (the relative difference in intensity between adipose and gluteus maximus) and the subcutaneous adipose characteristics (the relative difference in intensity between subcutaneous adipose under and surrounding the ischium) were compared to PrI history and subject characteristics.

**Results:** Participants with a history of PrIs had different subcutaneous fat (subQF) characteristics than participants without a history of PrIs. Specifically, they had significantly darker adipose under the ischium than surrounding the ischium (subQF effect size = 0.21) than participants without a history of PrIs (subQF effect size = 0.58). On the other hand, only when individuals with complete fat infiltration (n = 7) were excluded did individuals with PrI history have more fat infiltration than those without a PrI history. The presence of spasms (μ intramuscular adipose, 95% CI with spasms 0.642 [0.430, 0.855], without spasms 0.168 [−0.116, 0.452], *p* = 0.01) and fewer years using a wheelchair were associated with leaner muscle (Pearson Corr = −0.442, *p* = 0.003).

**Conclusion:** The results of the study suggest the hypothesis that changes in adipose tissue under the ischial tuberosity (presenting as darker SubQF) are associated with increased biomechanical risk for pressure injury. Further investigation of this hypothesis, and the role of intramuscular fat infiltration in PrI development, may help our understanding of PrI etiology. It may also lead to clinically useful diagnostic techniques that can identify changes in adipose and biomechanical risk to inform early preventative interventions.

## Introduction

Full-time wheelchair users are at a higher risk for pressure injuries (PrIs) due to continuous, high magnitude loading and lack of sensation (Bergstrom et al., 1995; Salzberg, et al., 1996). Disability categories with the highest PrI prevalence include paraplegia/quadriplegia, spina bifida, Alzheimer disease, hemiplegia, cerebral palsy, Parkinson disease, and multiple sclerosis (Sprigle et al., 2020). Previous research has identified many risk factors such as age, poor nutrition, and shearing, which significantly add to an individual’s PrI risk (Braden and Bergstrom, 1987; European Pressure Ulcer Advisory Panel, National Pressure Injury Advisory Panel and Pan Pacific Pressure Injury Alliance, 2019). Investigation into the role of adipose and its relationship to tissue tolerance and PrI risk has been limited, despite considerable evidence on the effect of adipose on an individual’s health (Garcia and Thomas 2006; Lemmer, Alvarado et al., 2019; Bogie, Schwartz et al., 2020).

There are multiple kinds of adipose depots, such as visceral, intramuscular, and subcutaneous, that differ in adipose characteristics. Intramuscular adipose tissue (IMAT) is the sum of the fat that lies underneath the fascia and between muscle groups and the fat that is infiltrated between and/or within the muscle fibers. Subcutaneous fat (SubQF), by definition, exists deep to the epidermis and dermis and represents the majority of body fat.

The majority of research about adipose is not focused on pressure injuries but on obesity, diabetes, and related disciplines. IMAT in obese and glucose-tolerant individuals has been found to be associated with decreased muscle quality and increased insulin resistance, which increases the risk for type 2 diabetes (Goodpaster, Thaete et al., 2000; Addison, Marcus et al., 2014). Like IMAT, previous research has shown that SubQF located in the abdominal region has been correlated with insulin resistance. However, in the gluteal–femoral region, the SubQF tissue has been shown to protect against metabolic dysregulation and insulin resistance (Patel and Abate 2013; Addison, Marcus et al., 2014). Additionally, lower body SubQF has been found to protect systemic glucose homeostasis and inhibit obesity-induced muscle pathophysiology (Booth, Magnuson et al., 2018). Previous research suggested that the protection might be due to fat storage capacity (Grundy 2015; Booth, Magnuson et al., 2018). As a result, this would increase blood flow, since an increased insulin resistance is correlated to decreased skin blood flow regulation (Snijder et al., 2005; Yim, Heshka et al., 2008; Liao, Burns et al., 2013; Bhattacharya and Mishra 2015; Lambadiari, Triantafyllou et al., 2015).

In early studies, IMAT has been found to be directly correlated with PrI history in individuals with a spinal cord injury (Ogawa, Lester et al., 2017; Lemmer, Alvarado et al., 2019). A follow-up study on individuals with spinal cord injuries found that IMAT was correlated with circulatory adipogenic and myogenic biomarkers (Bogie, Schwartz et al., 2020). Although IMAT is common, the mechanisms for its development and effects remain unknown. Therefore, understanding the individual characteristics that predict IMAT for a wheelchair user (e.g., age, years of immobility, spasticity, etc.) may inform the physiology behind the infiltration process. With this knowledge, future interventions may target improvement of muscle quality. Furthermore, it would be beneficial to confirm previous results regarding the relationship between IMAT and PrI development in a larger population of wheelchair users.

Contrary to the role of IMAT, a loss of subcutaneous fat (SubQF) has been shown to increase PrI risk (Garcia and Thomas 2006; Sonenblum, Seol et al., 2020). Obesity, exercise, and hypoxia can all change the characteristics of adipose (Alkhouli, Mansfield et al., 2013; Frayn and Karpe 2014; Lempesis et al., 2020). Furthermore, adipogenesis is a mechanosensitive process (Hara, Wakino et al., 2011; Levy, Enzer et al., 2012). Therefore, the constant loading experienced by wheelchair users who sit nearly 12 h/day (Sonenblum and Sprigle 2011; Sonenblum, Sprigle et al., 2012) has significant potential to change the characteristics of the SubQF of wheelchair users. To date, these changes have not been studied.

Biomechanical risk is an intrinsic characteristic of an individual’s soft tissue to deform in response to extrinsic applied forces, and it is associated with PrI risk (Sonenblum, Seol et al., 2020). Understanding biomechanical risk is critical for assessing PrI risk and optimizing PrI prevention, but changes that occur in SubQF and how they influence biomechanical risk have not yet been studied. Furthermore, understanding what subject characteristics are associated with change in SubQF characteristics may help identify factors that assist in PrI prevention.

This study sought to accomplish two goals: (1) to identify the relationship between adipose (SubQF and IMAT) characteristics and PrI history in wheelchair users and 2) to identify subject characteristics, including biomechanical risk, that are related to adipose (SubQF and IMAT) characteristics. Because the changes in adipose are poorly understood, we also investigated whether IMAT was associated with SubQF characteristics.

## Methods

### Subjects

Forty-three individuals who use wheelchairs as their primary mobility device were included in this exploratory secondary analysis. Participants were recruited from either the New York, Colorado, Georgia, or New Jersey area. This study was approved by institutional review boards at a primary research site and multiple clinical sites. All participants reviewed and signed the informed consent form as approved by their local institutional review board prior to participation in the study. Inclusion criteria required that the participants must have been using a wheelchair for at least 2 years and were able to remain stable while seated on flat foam in the MRI environment. If the participants had a current PrI, they could not be on restricted sitting time or be considered at risk from sitting on the test cushions or performing additional transfers. A subset of this population has been published previously in Sonenblum, Seol et al. (2020).

### Study Protocol

Each subject had their characteristics, such as age, body mass index (BMI), PrI history, and presence of spasticity, collected *via* a self-reported health form. Participants reported a PrI history if they had experienced at least one prior PrI at an ischium or in the sacral/coccygeal region, and at which location(s) they had experienced the PrIs. Participants’ buttocks were scanned while they sat in a FONAR UPRIGHT^®^ MRI. Since these scans were originally taken for the purpose of measuring seated tissue deformation, a FONAR UPRIGHT^®^ MRI was used, and T1-weighted (RF spoiled) 3D in-phase gradient echo scans were taken with participants seated. Scans had a 280-mm field of view with 3.0 mm contiguous slices in the sagittal plane. The field of view was centered on the right ischial tuberosity for most participants, unless participants had an active pressure ulcer on that side or any hardware that would cause an artifact in the MRI (such as a hip implant), in which case the left ischial tuberosity was the center of the field of view. Most participants were scanned while seated on an 18″ × 18″ cushion made with flat 3″ HR45 foam. However, five participants were not studied in this condition. Therefore, for this analysis, scans collected on a Matrx Vi (Invacare, n = 4), or Embrace (Permobil, n = 1) were used. Both the Embrace and Matrx Vi are contoured foam cushions, as opposed to flat foam, but they created a similar loaded condition to study the adipose characteristics of the buttocks.

Expert clinicians assisted with seating the subjects to have their pelvis in a neutral posture. The footrest was also adjusted to properly load the thighs and to keep the knees and hips close to 90° of flexion (Sonenblum, Seol et al., 2020).

An additional subject characteristic was measured with subjects seated on the same reference foam on which they were imaged. Compressible hip breadth was measured by measuring the bi-trochanteric distance twice: once with no tissue compression and once with maximum compression (i.e., until the tissue could not be compressed any farther), and the difference between the two was computed.

### Data Processing

#### MRI Image Processing

The raw DICOM scans were imported into MRI analysis software, AnalyzePro (AnalyzeDirect, Overland Park, KS), for review and segmentation of the pelvis, gluteus maximus, and the subcutaneous fat of the side of the buttocks included in the MRI. Trained researchers under the supervision of an experienced radiographer (Cathcart) performed the segmentations for a single slice of the MRI scans located at the peak of the ischial tuberosity in the sagittal plane. Skin was included within the subcutaneous fat segmentation when visible since the scan resolution did not allow for separate segmentation of the two. The region pixel intensities and the coordinate location points from the adipose and gluteus maximus segmentations from the sagittal slice were extracted and interpreted using MATLAB R2020a (MathWorks, Natick, MA).

Measures associated with biomechanical risk included bulk tissue thickness and sagittal radius of curvature, and their calculations have been described previously (Sonenblum et al., 2020). Briefly, bulk tissue thickness is defined as the average tissue thickness under the ischial tuberosity measured in an oblique plane in a region 50 mm long. The radius of curvature is computed in a 50-mm region centered at the peak of the ischial tuberosity in the sagittal plane.

Because the scans were taken with a planar surface coil (Quad-Z Planar) located in the axial plane under the wheelchair cushion, there was an image intensity gradient along an axis orthogonal to the coil, i.e., in the superior–inferior direction. Therefore, it was necessary to correct for this gradient to allow for accurate comparison between the gluteus maximus and adipose intensities when calculating intramuscular fat infiltration.

**FIGURE 1:**
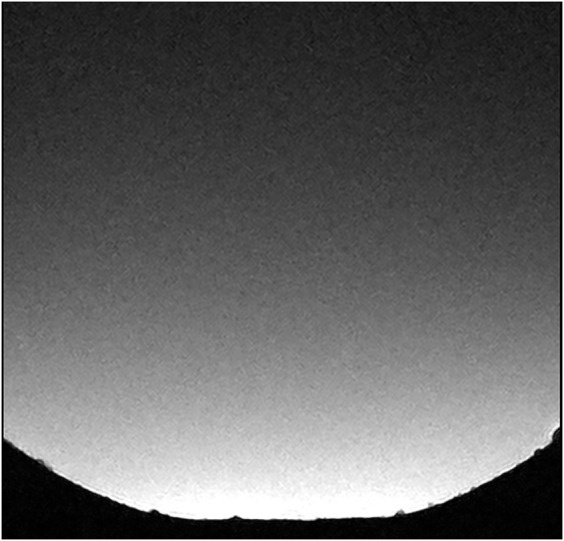
The control scan of a basketball filled with dilute nickel chloride that was used to find the power function (i.e., normalization curve) for normalizing the intensity gradient in scans of the buttocks.

To correct for the image intensity gradient, we used a control MRI scan of a basketball filled with dilute nickel chloride (to mimic human tissue) that was placed on top of the Quad-Z Planar coil at its center (Figure 1) with the same sequence parameters used to scan participants. Because the basketball was filled with a homogenous fluid, the smooth variation in pixel brightness from point to point (voxel to voxel) can be attributed to voxel distance from the conductive elements of the receiver coil. Therefore, a normalization curve was defined to represent the variation in intensity of the image using the power function through MATLAB’s Curve Fitting Tool as a function of distance across the vertical axis of the scan (the superior–inferior direction). Sagittal voxel intensities in the buttocks scans were corrected by subtracting the normalization curve from the original scan intensities.

#### Intramuscular Adipose Tissue

**FIGURE 2:**
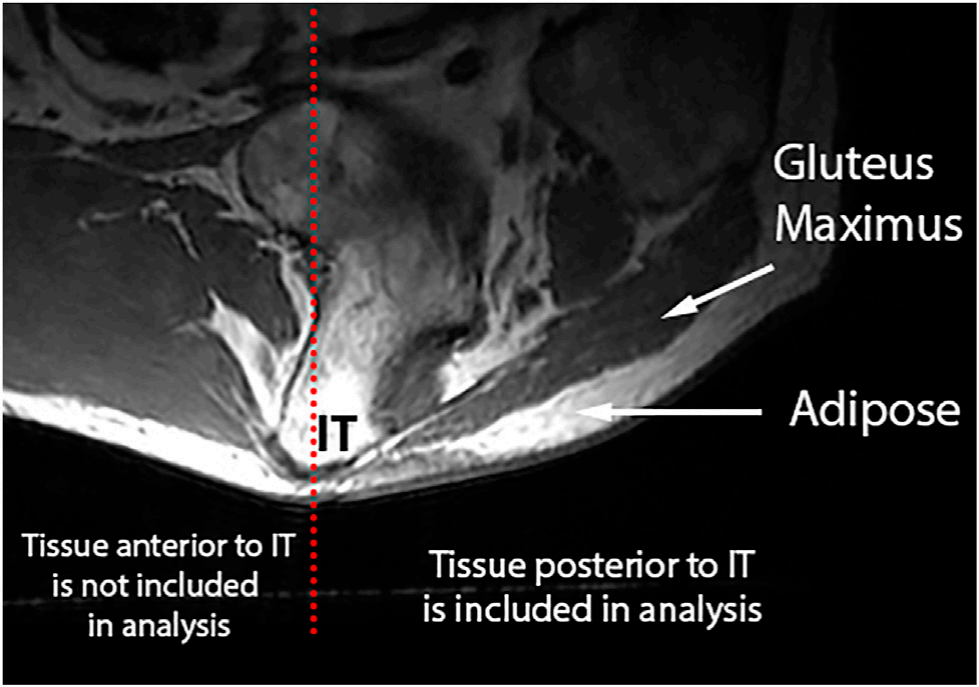
IMAT is calculated using the gluteus maximus and SubQF intensities posterior to the ischial tuberosity.

To study the IMAT within the gluteus maximus, which resides predominantly posterior to the gluteus maximus (Sonenblum, Seol et al., 2020), adipose included in IMAT analysis was constrained to the region posterior to the ischial tuberosity and inferior to the gluteus maximus (Figure 2).

Several approaches were considered to quantify IMAT in the gluteus maximus. Methods like the midpoint method and the Otsu Method (Gorgey, Ogawa et al., 2017; Ogawa, Lester et al., 2017) rely on identifying adipose and gluteus intensities within the gluteus maximus and computing a cutoff threshold. While this is effective in many cases, for subjects with very lean gluteus maximi (i.e., no IMAT) or fully adipose infiltrated muscle (i.e., no discernable muscle tissue), identifying separate tissue types and therefore an intensity cutoff threshold is not effective.

Instead, we reported an effect size for each participant. The IMAT effect size is a standardized magnitude of difference between the corrected intensities of the gluteus maximus and the SubQF inferior to the gluteus (Eq. 1). Higher IMAT effect sizes indicated less IMAT within the gluteus maximus, while values closer to zero or even negative IMAT effect sizes indicate significant amounts of IMAT in the gluteus maximus.

Equation 1. IMAT effect size is the relative difference in intensity between adipose and gluteus maximus.

$$\mathrm{IMAT}\;\mathrm{effect}\;\mathrm{size}=\frac{\mathrm{mean}\;\mathrm{intensity}\;\mathrm{of}\;\mathrm{the}\;\mathrm{adipose}-\mathrm{mean}\;\mathrm{intensity}\;\mathrm{of}\;\mathrm{the}\;\mathrm{gluteus}\;\mathrm{maximus}}{\mathrm{pooled}\;\mathrm{standard}\;\mathrm{deviation}\;\mathrm{of}\;\mathrm{intensities}\;\mathrm{in}\;\mathrm{both}\;\mathrm{tissues}}$$ (1)

This approach was correlated (Pearson Corr = −0.601, *p* = 0.003) with the midpoint method for participants not at the extremes of low or high IMAT (e.g., the interquartile range). Furthermore, MRI scans were subjectively categorized based on who had low, medium, and high fat infiltration, and the effect size accurately predicted these categorizations.

#### Subcutaneous Adipose (SubQF) Tissue

**FIGURE 3:**
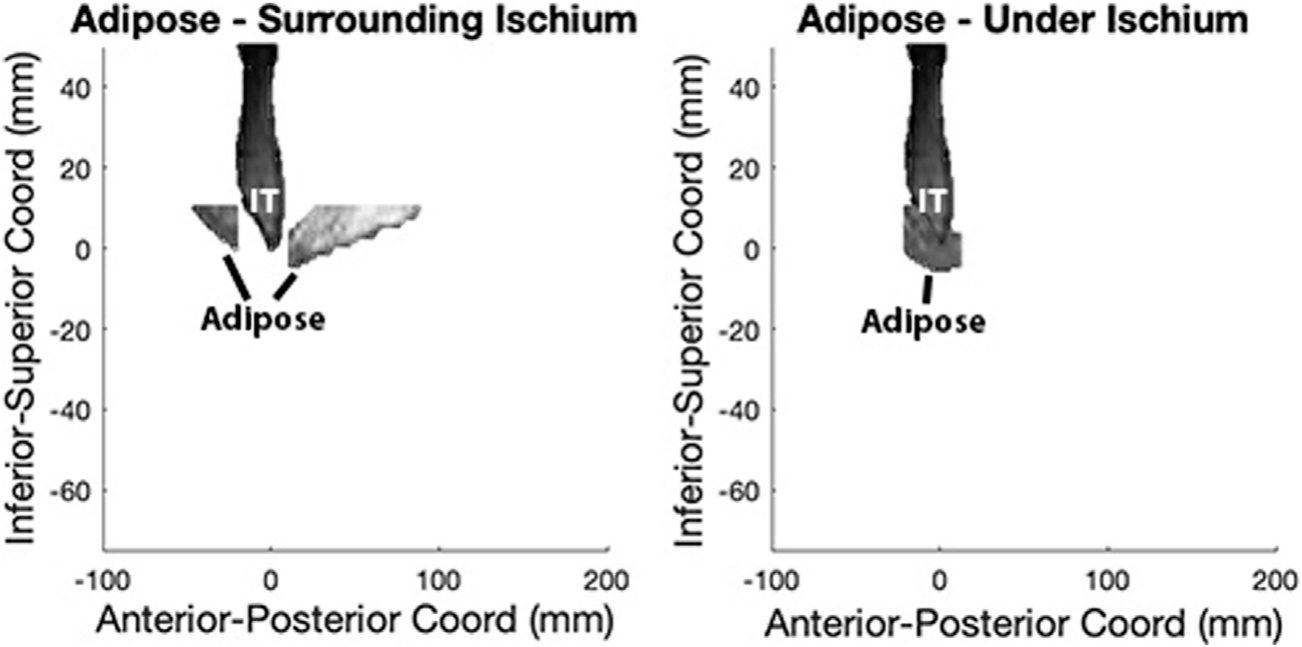
SubQF effect size is calculated using adipose under and surrounding the ischium in the sagittal plane.

Since the adipose under the pelvis is the most susceptible area to PrIs, we evaluated the SubQF under the ischium and compared it with the segmented SubQF surrounding the ischium in the sagittal plane (Figure 3). First, we located all SubQF inferior to a location 10 mm superior to the peak of the ischium. Then, we further divided this segment of SubQF into two regions: 1) under the ischium included 5 mm anterior and posterior of the ischium from 10 mm superior to the most inferior aspect of the ischial tuberosity, and 2) surrounding the ischium included regions anterior and posterior to the region under the ischium. This constrained the adipose included in analysis and avoided unintentional inclusion of visceral adipose. To describe the characteristics of the SubQF for each individual, we quantified the standardized magnitude of the difference in intensity under the ischium versus surrounding the ischium using the effect size (Eq. 2).

Equation 2. SubQF effect size is the relative difference in intensity between SubQF under and surrounding the ischium.

$$\mathrm{SubQF}\;\mathrm{effect}\;\mathrm{size}=\frac{\mathrm{mean}\;\mathrm{intensity}\;\mathrm{of}\;\mathrm{adipose}\;\mathrm{surrounding}\;\mathrm{the}\;\mathrm{ischium}-\mathrm{mean}\;\mathrm{intensity}\;\mathrm{of}\;\mathrm{adipose}\;\mathrm{under}\;\mathrm{the}\;\mathrm{ischium}}{\mathrm{pooled}\;\mathrm{standard}\;\mathrm{deviation}\;\mathrm{of}\;\mathrm{intensities}\;\mathrm{in}\;\mathrm{both}\;\mathrm{regions}}$$ (2)

### Data Analysis

The goals of this study were to identify the relationship between adipose characteristics and PrI history in wheelchair users and to identify subject characteristics that are related to adipose characteristics. To accomplish these goals, one-way ANOVA tests were run comparing adipose characteristics across individuals with and without a history of PrIs. Statistics were computed using Minitab 18.1. Four subject characteristics were considered: BMI, years using a wheelchair, presence of spasticity, and compressible hip breadth. All but hip breadth were assessed *via* self-report. The relationship between adipose characteristics (SubQF effect size and IMAT effect size) and continuous characteristics—BMI, years using a wheelchair, and compressible hip breadth—were investigated with a correlation, while the presence of spasticity was studied with a one-way ANOVA.

To understand if adipose characteristics played a role in deformation of the seated buttocks, correlations were calculated between adipose characteristics (i.e., SubQF effect size and IMAT effect size) and bulk tissue thickness and sagittal radius of curvature. Finally, a correlation was calculated to investigate a relationship between subcutaneous adipose and intramuscular adipose characteristics.

## Results

### Participants

Participants were predominantly men (81%) with spinal cord injuries (84%) (Table 1). The remaining participants had spinal cord disorders such as spinal bifida (n = 4) and spinal cord stroke (n = 1), while one participant had multiple sclerosis and one had fronto-temporal degeneration. They ranged in age from 18 to 73 years old and had between 2 and 46 years of experience using a wheelchair as their primary mobility device. Additional characteristics are presented in Table 1 for all participants where responses and/or measurements were available.

**TABLE 1:** Participant characteristics.

	All		WC user No Hx (n = 21)		WC user PrI Hx (n = 22)	
	Mean (SD)	Median (min–max)	Mean (SD)	Median (min–max)	Mean (SD)	Median (min–max)
Age (years, n = 43)	46.8 (11.3)	47.0 (18.0–73.0)	44.8 (13.5)	46.0 (18.0–73.0)	48.8 (8.7)	48.5 (34.0–66.0)
BMI (n = 43)	23.9 (4.8)	23.1 (14.6–34.5)	24.5 (5.0)	23.9 (14.6–34.5)	23.3 (4.6)	21.5 (14.9–33.5)
Years using wheelchair (n = 43)	15.3 (12.2)	10.0 (2.0–46.0)	10.7 (9.3)	6.5 (2.0–37.0)	19.6 (13.3)	16.3 (3.0–46.0)
Compressible hip breadth (inches) (n = 39)	1.4 (0.8)	1.3 (0.3–3.0)	1.7 (0.8)	1.8 (0.5–3.0)	1.1 (0.8)	0.8 (0.3–3.0)
Sex	N	%	N	%	N	%
Female	8	19%	6	29%	2	9%
Male	35	81%	15	71%	20	91%
Diagnosis						
SCI	36	84%	17	81%	19	86%
Other	7	6%	4	19%	3	14%
Injury completeness (n = 41)						
Complete	22	54%	10	48%	12	60%
Incomplete	19	46%	11	52%	8	40%
Spasms (n = 38)						
Yes	24	63%	10	56%	14	70%
No	14	37%	8	44%	6	30%
Race						
Asian American	1	2%	1	5%		
Black/African American	2	5%	1	5%	1	5%
White	33	77%	16	76%	17	77%
Hispanic or Latino	5	12%	2	9%	3	14%
Two or More Races	1	2%			1	5%
Other	1	2%	1	5%	74">	86">

### Adipose Characteristics and Pressure Injuries

#### Subcutaneous Adipose

**FIGURE 4:**
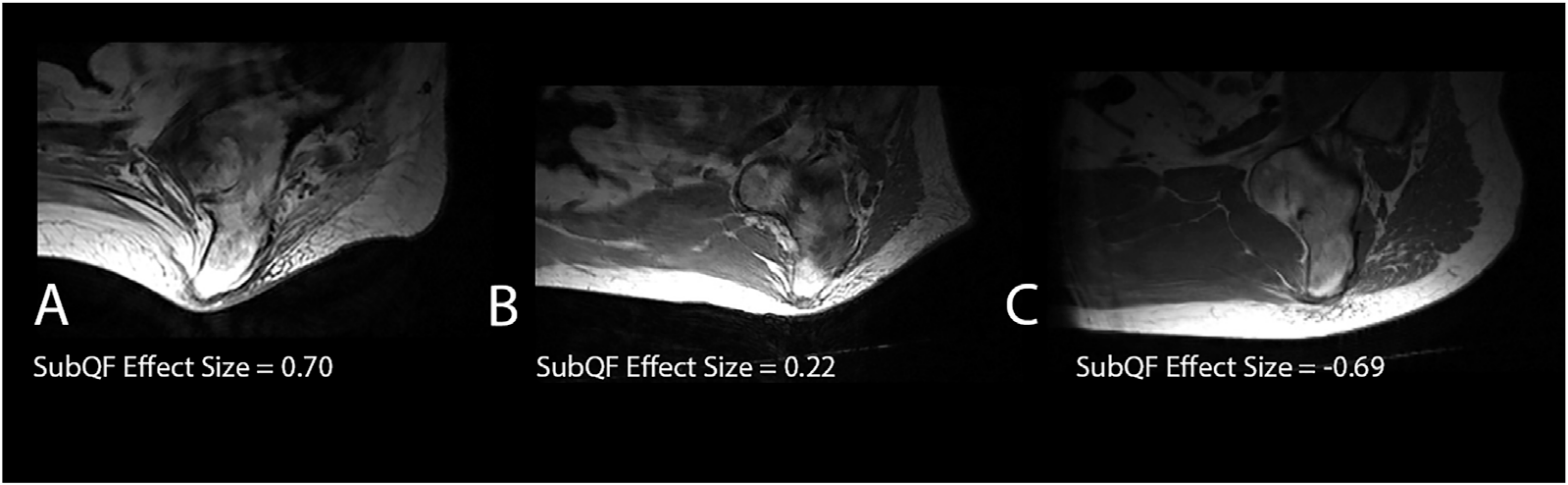
Examples of subjects with different SubQF effect sizes. Subject A had a SubQF effect size of 0.7, indicating darker adipose under the ischium than surrounding the ischium, while subject B had a SubQF effect size of 0.22, indicating adipose under the ischium was only slightly darker than surrounding ischium. Subject C (SubQF effect size of −0.69) actually had brighter adipose under the ischium.

Characteristics of the adipose tissue underneath the ischium were different than the surrounding adipose in some people, leading to larger SubQF effect sizes. Larger, positive SubQF effect sizes indicate that the adipose under the ischium is darker than surrounding adipose (Table 2; Figure 4). Greater negative SubQF effect sizes indicate that the tissue under the ischium is brighter than the tissue surrounding the ischium.

**TABLE 2:** Adipose characteristics.

Variable	All (n = 43)	No PrI (n = 21)	Yes PrI (n = 22)	p-value
	Mean (SD)	Mean (SD)	Mean (SD)	
Adipose intensity surrounding the ischium	2,022 (671)	2,019 (657)	2,026 (700)	0.974
Adipose intensity under the ischium	1,707 (783)	1,873 (842)	1,548 (704)	0.176
SubQF effect size	0.40 (0.53)	0.21 (0.56)	0.58 (0.45)	0.022
Adipose intensity posterior to the ischium	1,517 (387)	1,586 (388)	1,451 (383)	0.258
Gluteus Maximus Intensity	1,324 (306)	1,313 (239)	1,334 (364)	0.831
IMAT effect size	0.50 (0.55)	0.59 (0.60)	0.41 (0.51)	0.280
Average bulk thickness (mm)	14.8 (6.1)	17.1 (7.1)	12.5 (3.9)	0.012
Sagittal radius of curvature (mm)	83.7 (38.1)	93.9 (44.7)	73.6 (27.5)	0.085

Participants with a history of PrIs had a significantly greater SubQF effect size than participants without a history of PrIs (difference in effect size μ [95% CI] = −0.370 [−0.684, −0.555], *p* = 0.022).

**FIGURE 5:**
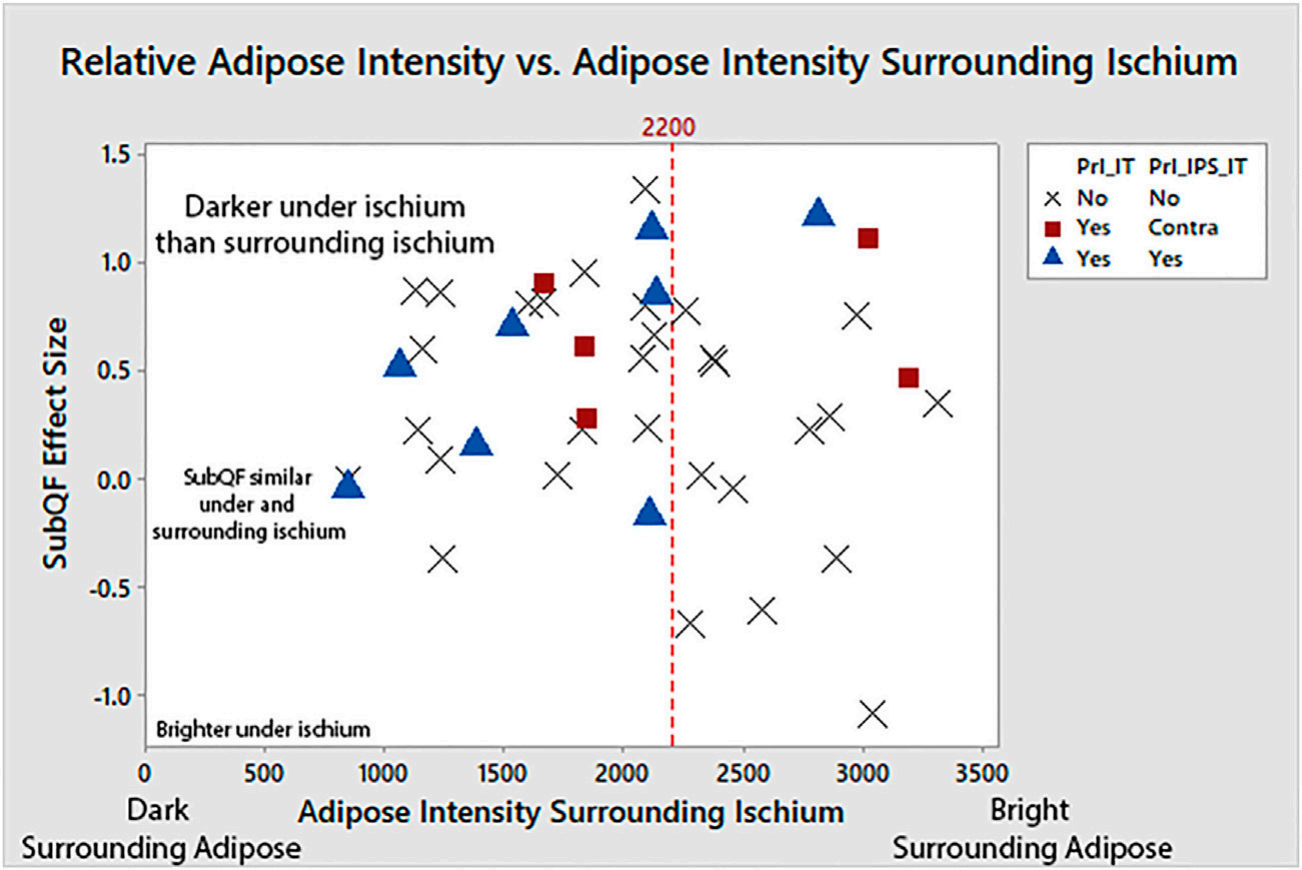
Adipose under the ischium was darker than surrounding adipose (larger SubQF effect size) for people with a history of PrIs, indicating changes to adipose characteristics under the ischium. People with ischial PrIs were also less likely to have bright surrounding adipose.

A closer look at participants with a history of ischial PrIs (i.e., 12 of the overall 22 participants with pelvic PrIs, excluding the 10 that had sacral/coccygeal PrI) revealed darker tissue surrounding the ischium (Figure 5). Among participants with bright adipose surrounding the ischium (e.g., >2,200), only three had a history of ischial PrIs. Specifically, participants with a history of PrIs had darker adipose under the ischium relative to the surrounding ischium.

#### Intramuscular Adipose Tissue

**FIGURE 6:**
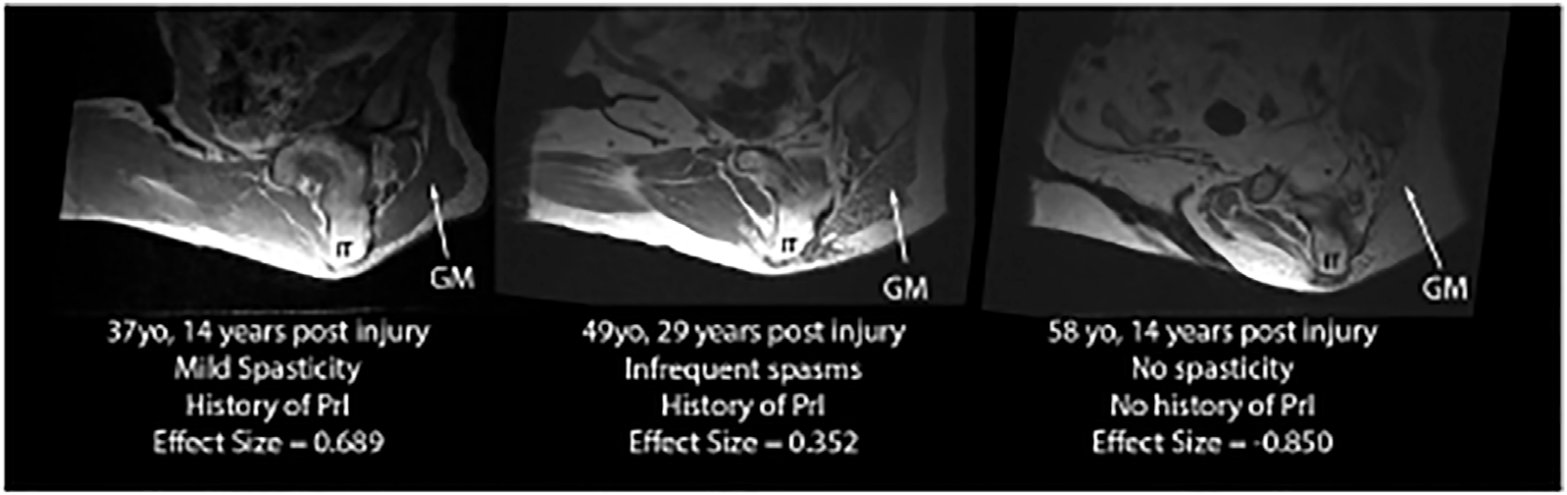
Examples of IMAT effect sizes varying from lean (IMAT effect size = 0.689) to full fat infiltration (IMAT effect size = −0.850).

Intramuscular adipose tissue varied, including very lean subjects with high effect sizes, such as the example on the left in Figure 6 (IMAT effect size = 0.689, gluteus maximus intensity darker than SubQF intensity), subjects with some IMAT present in the gluteus maximus (IMAT effect sizes >0), and subjects with complete fat infiltration. For these subjects with no muscle visible given all the adipose infiltration, IMAT effect size varied from −0.275 to −1.059 because the gluteus maximus intensity was brighter than the SubQF intensity (e.g., Figure 6, right).

One-way ANOVA showed that there was no relationship between fat infiltration and PrI history (Table 2). However, when individuals with complete fat infiltration (n = 7) (i.e., their muscle is indistinguishable from the adipose) were excluded, individuals with PrI history had more fat infiltration than those without a PrI history (IMAT effect size with PrIs n = 19 and µ, 95% CI = 0.56 [0.428, 0.693] vs. without n = 17 and µ = 0.86 [0.716, 0.997], difference in IMAT effect size µ, 95% CI = 0.29 [0.102, 0.471], *p* = 0.004).

### Adipose Characteristics and Individual Characteristics

#### Subcutaneous Adipose

Patient characteristics were not predictive of subcutaneous adipose characteristics. Specifically, there was no significant correlation between the differences in adipose inferior to the ischium versus surrounding the ischium (SubQF effect size) and BMI, years using wheelchair, or compressible hip breadth. Furthermore, one-way ANOVA tests revealed no differences in subcutaneous effect sizes according to the presence of spasms.

#### Intramuscular Adipose Tissue

**FIGURE 7:**
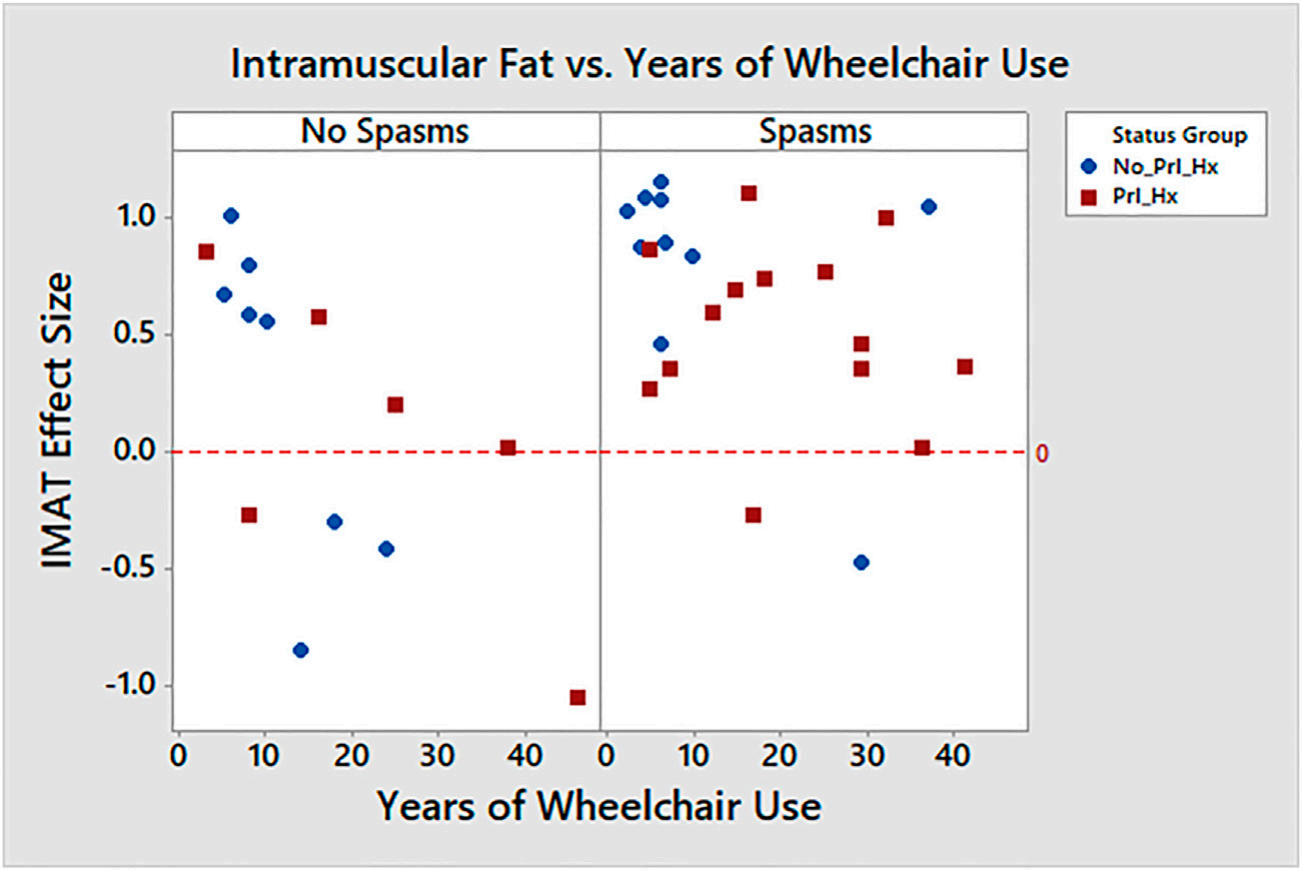
Intramuscular fat vs. years of wheelchair use per spasticity.

Some patient characteristics were associated with intramuscular adipose (Figure 7). Specifically, the presence of spasms was associated with a greater IMAT effect size, or leaner muscle (μ, 95% CI with spasms 0.642 [0.430, 0.855], without spasms 0.168 [−0.116, 0.452], *p* = 0.01). BMI was not associated with intramuscular adipose (Pearson Corr = −0.148, *p* = 0.339) nor was compressible hip breadth (Pearson Corr = −0.193, *p* = 0.239), but years using a wheelchair was negatively correlated with IMAT effect size (Pearson Corr = −0.442, *p* = 0.003), suggesting more fat infiltration with more years in the wheelchair.

### Adipose Characteristics and Biomechanical Risk

#### Subcutaneous Adipose

Subcutaneous effect size was negatively correlated with tissue thickness (Pearson Corr = −0.489, *p* < 0.01) and sagittal radius of curvature = −0.564, *p* < 0.01). That is, as SubQF effect size goes up (darker adipose under IT), tissue thickness goes down, and the radius of curvature goes down or the shape becomes more peaked. In other words, darker adipose under the IT is associated with a higher biomechanical risk.

#### Intramuscular Adipose

Intramuscular adipose (IMAT effect size) was not related to either tissue thickness or radius of curvature of the seated buttocks (Pearson Corr = 0.092 and = −0.073 respectively).

### Intramuscular Adipose vs. Subcutaneous Adipose

SubQF effect size was not related to IMAT effect size (Pearson Corr = −0.100).

## Discussion

This study is the first to integrate an assessment of both subcutaneous and intramuscular adipose in wheelchair users and to assess how adipose relates both to PrI risk and biomechanical risk (i.e., the tissue response to loading).

Tissue with darker subcutaneous adipose under the IT compared with surrounding adipose measured with an MRI was associated with a higher biomechanical risk when seated. These individuals were also more likely to have a history of PrIs, but not necessarily at the ipsilateral ischial tuberosity that was studied. The relationship between adipose and tissue tolerance likely evolves over time in response to load. Buttocks with good structural integrity and low biomechanical risk, such as the buttocks of an able-bodied adult, provide ample protection from development of PrIs.

There are a number of physiological reasons why changes to adipose characteristics might occur over time for wheelchair users and consequently influence tissue tolerance and biomechanical risk. Studies have shown that exposure to mild, prolonged hypoxia increases fat storage and decreases proinflammatory gene expression. In contrast, acute exposure to severe hypoxia decreases fat storage and increases proinflammatory expression (Lempesis et al., 2020). Loading over time will also bring about changes to the mechanical structure of the subcutaneous adipose (Edsberg, Cutway et al., 2000; Edsberg, Natiella et al., 2001). For example, omental adipose has a higher proportion of fibrous proteins than subcutaneous adipose (Alkhouli et al., 2013). Thus, if the tissue were to differentiate in response to load, the distribution of fibrous proteins might change. Additionally, the extracellular matrix of adipose is composed primarily of collagen, yet elastin provides greater distensibility (Alkhouli et al., 2013). Therefore, a change in the distribution of collagen and elastin would result in a change in mechanical properties and biomechanical risk. Finally, lipid formation is known to change in response to load (Hara et al., 2011), and this may also affect the mechanical properties and, thus, the biomechanical risk (Ben-Or Frank et al., 2015).

Clinically, the association between adipose characteristics and biomechanical risk presents an opportunity both for assessment and intervention. For years, clinicians have been assessing tissue quality *via* palpation. The results of this study suggest that palpation may be augmented with an objective assessment of SubQF adipose quality to understand biomechanical risk and tissue tolerance. As MRI is not practical for regular clinical use, this would require clinically viable tools to assess SubQF quality, whether *via* imaging, assessment of mechanical properties, or an alternative approach. Furthermore, if changes in mechanical structure of the adipose increases risk, improving the SubQF quality could be an interventional goal. Lastly, combined with the finding that there is little gluteus maximus coverage of the ischium while seated (Sonenblum et al., 2020), these results provide further evidence that adipose plays an important role in PrI development.

In terms of intramuscular adipose tissue, our results contrasted with the studies of Lemmer, Alvarado et al. (2019)and Wu and Bogie (2013). Our study found that there was no relationship between the amount of fat infiltration and PrI history, which was unanticipated. It is possible that when studying such small sample sizes as done in Lemmer, Alvarado et al. (2019) (n = 38, but only n = 11 did not have a PrI history) and Wu and Bogie (2013) (n = 10 wheelchair users), IMAT was serving as a proxy for other effects such as time since injury, or in the present study, time since injury could be confounding the effect of IMAT. It is also worth noting that the methodology used to measure IMAT was different between these studies. Lemmer, Alvarado et al., (2019) and Wu and Bogie (2013) used CT scans to measure IMAT, which allowed for a more precise calculation of the ratio of adipose and muscle than is possible using MRI. On the other hand, CT scans come with increased radiation exposure and a supine posture that was undesirable for the present data collection approach.

However, when the completely fat-infiltrated subjects (IMAT effect size <0, or gluteus maximus intensity > adipose intensity) were excluded in our study, subjects with a higher amount of fat infiltration were more likely to have a history of PrI. Fat infiltration increases insulin resistance (Kalyani et al., 2014), which may be associated with decreased microvascular flow to the skeletal muscle and could explain the increased risk in this subset of the population. The difference in findings between people with partial fat infiltration and full fat infiltration may indicate that people who are fully fat infiltrated may have a different pathology of fat infiltration, making fat infiltration contribute less to their risk for PrI. For example, one participant experienced fat infiltration secondary to chemotherapy. Another possibility is that the properties and oxygen demand of the fully infiltrated muscle are different than that of the partially infiltrated muscle. Previous research has shown that in the abdominal region, subcutaneous fat has been correlated with insulin resistance (Patel and Abate, 2013). However, in the gluteal–femoral region, subcutaneous adipose tissue has been shown to increase insulin, which would increase blood flow (Snijder et al., 2005; Yim et al., 2008; Lambadiari et al., 2015). If the IMAT properties conformed to properties similar to SubQF upon full fat infiltration, then this could explain a change in perfusion and risk. At the same time, oxygen requirements are lower with enlarged fat cells, which may be the phenotype present in a fully infiltrated muscle (Frayn and Karpe, 2014). However, additional research would need to be conducted to confirm these new hypotheses.

Since BMI is a common method used to measure an individual’s body fat, one would expect that the amount of fat infiltration and BMI would be directly correlated. However, there was no relationship between fat infiltration and BMI. This is consistent with Bogie et al. (2020). Time since injury is a much stronger predictor of IMAT, suggesting that the process occurs at some point after injury, likely as a result of disuse and atrophy, in response to myogenic and adipogenic markers identified in (Bogie et al., 2020). BMI (i.e., body weight and height) was self-reported in this study, so the accuracy of BMI values is unknown. However, our findings were similar when analyzed as SCI-adjusted BMI category, which would be less sensitive to precise weight measurements.

### Limitations

The MRI scans used in this study were not optimized for studying intramuscular or SubQF adipose. There are imaging protocols that would provide more detailed information about the adipose, such as a Dixon protocol. Furthermore, the characteristics and etiology of the darker presentation of SubQF observed would be more thoroughly investigated using histology and mechanical testing as opposed to MRI. However, we believe that the results of this investigation provide important preliminary results to drive future work, in which histology and mechanical testing will relate the imaging results to specific changes in adipose characteristics.

While this study explored a large group of individuals with and without PrI history, there was a difference in the years of wheelchair use between the two groups that may have impacted IMAT analysis. Additional factors and comorbidities, such as chemotherapy or diabetes, were also not analyzed but may have influenced IMAT. Further studies that will include longitudinal analysis and consideration of additional factors and comorbidities would be beneficial to better understand the natural history of fat infiltration.

The few participants who used a wheelchair for reasons other than a spinal cord injury may expect a different adipose and muscle presentation given the differences in autonomic nervous system function. However, variability in nervous system function among individuals with SCI also exists, and this is best addressed by studying a larger population.

## Conclusion

The results of the study suggest a hypothesis that changes in adipose tissue under the ischial tuberosity are associated with increased biomechanical risk for pressure injury. Further investigation of this hypothesis is warranted, including histology to understand the specific changes in tissue characteristic associated with darker fat, mechanical testing to identify changes in mechanical properties, and measurement of blood flow responses in regions with dark adipose. Further investigation of this hypothesis may lead to clinically useful diagnostic techniques that can identify changes in adipose and biomechanical risk to inform early preventative interventions.

## Data Availability

The raw data supporting the conclusions of this article will be made available by the authors, without undue reservation.
